# Some Account of a Fever Produced by the Decomposition of Potatoes

**Published:** 1853-02

**Authors:** F. K. Bailey

**Affiliations:** Almont, Lapeer Co., Mich.


THE NORTH-WESTERN

MEDICAL AND SURGICAL JOURNAL.

NEW SERIES.

VOL. I. FEBRUARY, 1853. No. 10.

### ORIGINAL COMMUNICATIONS.

ART. I.—*Some Account of a Fever produced by the Decomposition of Potatoes.* By F. K. BAILEY, M.D.

No subject connected with medical science has excited more interest than that of fevers. The only apology for the appearance of this article, is the thought that it may add a little to the stock of facts in reference to fever produced by local causes.

It is proposed, in this communication, to give a description, as well as may be, of a disease which commenced in this place, in the Autumn of 1846. Up to this time, from the first settlement of the town, the diseases in this region were of the same character as those usually met with in newly-settled places in the West. The country is generally level, and well timbered with beach, maple, oak, &c.

During the Summer of 1846, there was erected in this village a large building, to be used in the manufacture of potatoe starch. The farmers, within five miles in each direction, contracted to produce potatoes, and draw them to the factory. As soon as the middle of October, there were delivered and deposited in one reservoir, about thirty thousand bushels. The machinery for grating not being in readiness as soon as was expected, this amount of potatoes laid some two or three weeks. The weather being warm, the whole mass soon began to rot. A small proportion of them were diseased when drawn, but they were generally in a sound condition. As soon as it could be done, the pile was overhauled, and not far from twenty thousand bushels removed into the yard in front of the mill. This mass was some three or four rods in extent each way, and four to five feet deep in the centre. The smell emanating from this offensive heap of vegetable matter was almost intolerable.

The fevers during the Summer and the first of the Fall months, had been of a milder type than usual, yielding to proper treatment in a few days. During the months of November and December, a typhoid tendency could be perceived in every case that occurred. It was evident that some modifying cause was operating. One man, who was engaged in removing potatoes from the mill, was attacked with a remitting fever; but about the seventh day, congestion of the brain set in, and he soon died. This case will be alluded to again.

Another case was that of a young lady, who had spent the Summer some six miles from the village, in a neighborhood where fevers of the common type were prevalent, and had during the time an attack of remittent, but partially recovered before returning home. In the fore part of November she was taken with a relapse, in which the disease assumed a typhoid form. This case will also be referred to again.

During the fore part of December many cases of fever occurred, assuming a continued form, and none of the common type. As I took no notes at the time, it will be impossible to give in detail all the appearances in any one case, but will endeavor to describe, in general, most of the symptoms characterising a majority of the cases.

The disease seldom commenced abruptly with a chill, but the patient complained of an uneasy sensation in the head, back, and limbs. Many, in describing their feelings, said they thought they should have the ague. There were languor and lassitude, no distinct chilliness, but an inclination to draw up to the fire, and an indisposition to exertion either physical or mental. The tongue was found to be coated with a whitish fur, there was a disagreeable taste in the mouth, and the breath was fœtid. The state of things just described, continued sometimes a week; but generally the disease became confirmed in five or six days. Usually, at the expiration of this forming stage, a chill more or less distinct would usher in the disease, after which no more coldness would be experienced. At other times, the alternate cold and heat would gradually give place to continued heat and excitement, rendering it difficult to ascertain the exact time to date the outset.

The pulse was frequent, with an occasional exception ranging from 100 to 140, or more, in a minute. The respiration was also increased in frequency, in about the same ratio as that of the pulse.

The functions of the stomach were not much deranged, beyond an impairment of the appetite. There was generally a looseness of the bowels, or an increased susceptibility to the action of laxatives. Sometimes the alvine evacuations were natural in frequency, with but little appearance of diarrhœa. Hæmorrhage from the nose was a frequent occurrence, in cases attended with increased redness of the face, and headache. The pulse was generally uniform in frequency during the twenty-four hours, although there was occasionally a remission in the morning and sometimes in the evening, and even one in the night.

The muscular strength, frequently, not only in the milder cases, but also in some of those of a graver form, was not as much impaired as in our common fevers. With the exception of the head, no part was severely affected by pain. A tendency to drowsiness was common, more or less, in every case; at times, however, there was so much restlessness as to prevent sleep. There was more or less tenderness on pressure over the epigastrium and the right iliac region. A tympanitic state of the abdomen was common, varying according to the state of the alimentary canal. The tongue, as the disease advanced, became more coated at the base, but red at the tip, which would sometimes feel a little sore. Thirst was not intense, but there was a dryness in the mouth and fauces, requiring a small draught of water at short intervals.

The skin was generally dry and hotter than natural, but sometimes covered with a cool sweat. There was a peculiar pallid appearance to the countenance, with an extremely anxious look. The urine was not always diminished in quantity, but generally redder in color, and, after standing a short time, emitted an offensive odor.

The above are many of the appearances seen during the first seven days after the accession of the disease. On the eighth day, however, there was found to be an aggravation of all the symptoms which had characterised the first period. The patient became more drowsy. The tongue became dry, or darker colored, or deep red. Heat and dryness of the skin increased, or in cases attended at first with moisture, the sweating was more profuse. Pulse still frequent, and quicker in its beat. Patient weaker, and less inclined to exertion. Frequently complained of no pain, and on being asked how he felt, would reply, "Better," or "Not much sick." Less inclined to take nutriment, and calls for drink more seldom, not so much from absence of thirst, as a want of appreciation of his condition. Distended state of the abdomen continues, but in an aggravated degree; and, in cases, attended at the outset with looseness, the diarrhœa becomes frequent and troublesome. The urine darker colored and more scanty. Seldom any vomiting or even nausea. Subsultus commences in the worst cases, and sometimes spasmodic contractions in the body and limbs. Delirium or stupor now supervene. Deafness is almost an unfailing attendant, but occasionally the hearing was morbidly acute.

In many cases the rose-colored eruption made its appearance upon the neck and other parts of the body, about the tenth day, but it was not an unfailing attendant.

With these appearances, we found the patient at the close of the second week from the attack. In many cases, on or near the fourteenth day a crisis would take place, the symptoms becoming more favorable, and convalescence commencing from that time. If the disease continued longer than two weeks, all the appearances manifested during the preceding periods were still aggravated. The stupor was increased. The tongue still was dry, dark-colored, and cracked. It was tremulous when protruded; and it was often difficult, or even impossible, to get that organ outside the lips. Sordes began to collect upon the lips and gums. In some cases the "calor mordax" could be distinguished, and in others the surface was covered with a cold clammy sweat, communicating a sensation to the hand similar to that experienced when a dog's nose is thrust against it. Respiration difficult and sometimes stertorous. Patient complained of a ringing or buzzing in the ears. The pulse still frequent, but more feeble. There was picking of the bed-clothes, and grasping at imaginary objects in the room above the bed. Extreme muscular debility, the patient inclined to lie upon the back, and disposed to slide towards the foot of the bed. Diarrhœa continues, and the stools darker-colored and grumous, having the appearance of dissolved blood, or, os aften remarked, looked like "beef brine." Abdomen still distended, and feeling much like a lump of dough. In the worst cases, the rectum and bladder were evacuated involuntarily. It may be mentioned here, that in some cases, at the close of the second period, there would be a partial change for the better; the white coat would leave the tongue; but, instead of appearing natural, that organ would become very red, and present a glazed, shining appearance, which in two or three days would change to a dark color; sordes then would collect, and other symptoms before-described make their appearance. At the close of the third week, in the favorable cases, was generally observed an amelioration of all the symptoms. The tongue began to assume a moist and natural appearance. The pulse still frequent, but less quick and more full and soft. Diarrhœa lessened or entirely stopped, and the stools more healthy in appearance. The skin, if heretofore dry, became more moist and soft, and the urine more copious and less red and dark-colored. At this stage, in some cases, sloughs and deep-seated abscesses would appear in different parts of the body, rendering convalescence slow and tedious.

In the unfavorable cases, however, no change for the better could be seen at this time. The pulse became more feeble, often intermitting. The whole surface covered with a cool, clammy sweat. Subsultus almost incessant, and hiccough distressingly frequent. The patient would sink gradually away, with little or no pain, and die like one going to sleep.

In cases where the disease terminated fatally, before the time that crisis would occur in that particular type, the patient would suffer the most excruciating pain; but if, on the other hand, it was protracted beyond the critical period, there would be a gradual sinking of the vital powers, attended with little or no suffering. Sometimes at the commencement of the fourth week, instead of a complete crisis, there only would be a partial change for the better.

Some of the morbid appearances would continue until the twenty-eighth day, when a complete crisis would occur, and the patient seem to awake from a long sleep, unconscious of how or where he had been during the course of the disease.

In the severer cases, the patient, on recovery, was unable to call to mind anything that occurred during his sickness. All was a blank which he was ever afterwards unable to fill up in his memory. The hair, in every case, I think, whether severe or mild, would fall off in the course of two months, leaving the scalp, in some instances, bald.

I have given a description of most of the morbid appearances observed in a majority of the cases that occurred during the Winter of 1846 and 1847. Taking the cases collectively, there was every grade of type and every degree of severity. Some were so mild, that all the variation from a healthy state was a slight feeling of debility; clean but somewhat red tongue; bowels slightly deranged; occasionally inactive, but generally somewhat loose, although not sufficient to demand medication. Some could sit in a chair one half of the day, and kept the bed more because the head felt better when in a recumbent position than from a sense of debility. In such cases there was some appetite and but little thirst. The pulse in this grade was uniformly more frequent than natural, being from 100 to 115 in a minute. The crisis occurred as soon as the fourteenth day, and frequently sooner.

Another grade was still more severe in all the appearances. The patient could sit up but a few minutes at a time. Tongue slightly coated, or red and dry. Some epigastric tenderness, and diarrhœa would supervene as soon as the tenth day, from which time until the twenty-first day the disease would progress steadily. Then a crisis would take place, and the patient begin to improve.

A third variety was still more severe. All the symptoms that made their appearance during the middle or last stages of the other grades, were seen in their severity during the first days of this. The most vigorous plan of treatment was requisite, and great watchfulness called for on the part of physician and nurse to control the attendants upon its daily progress.

The duration of the disease in question was variable. As stated above, it would terminate sometimes as early as the seventh or ninth day; again on the fourteenth, twenty-first, and twenty-eighth days. The uniformity with which the disease terminated depended upon whether there was any organic lesion. If there was none, the crisis was pretty sure to occur on the mentioned days, according to the grade of type. The instances were numerous, in which the disease was protracted indefinitely, by reason of such complications. In one instance there was peritoneal inflammation which confined the subject to the bed twelve weeks. In another, there was such a complete exhaustion, both mental and physical, that the sufferer remained for weeks in so imbecile a state as to forget everything she ever knew, even her own identity. Her faculties returned gradually, and she has since enjoyed perfect health.

In review, then, we have the following synopsis of morbid appearances, which may be considered as characteristic of the disease, we are attempting to describe:—

1. The pulse was frequent and quick. With an occasional exception, it was not less than 100 in a minute, and frequently going as high as 140 or 150 in cases that recovered. It was sometimes full, but always easily compressed beneath the finger. In the worst cases, and in feeble constitutions, it was sometimes found to intermit, which was usually an unfavorable symptom.

2. The breathing was hurried, frequently so much so as to occasion panting.

3. Morbid sweating was common, and was considered indicative of danger.

4. Diarrhœa was almost unfailing, and in many instances very excessive. Sometimes its existence was coeval with the attack, but frequently induced by the action of cathartics taken by the patient before calling medical aid, which developed the disease, that otherwise might not have occurred.

5. Tympanites was an attendant generally where there was diarrhœa, but sometimes when the bowels were torpid.

6. Cough was common, and frequently the first symptom complained of. The patient would seem to have a cold, cough a little for a day or two, when other symptoms supervened, and the disease was established.

7. Hæmorrhage from the bowels occurred in some cases, to such an extent as to exhaust the patient in a few hours. Sometimes the blood flowed unmixed, but commonly attended by morbid secretions.

8. The nervous system was in a marked degree affected in every case, more or less. Subsultus was common in the comparatively mild cases—worse, however, in persons of a strictly nervous temperament. Inability to confine the attention, indifference to surrounding objects and their own condition was apparent. But little solicitude was manifested in respect to recovery.

9. Delirium was common in the most cases, in some of which the patient would be almost uncontrollable. In a majority of cases the patient was so stupid, that it was difficult to obtain a correct answer to an inquiry. He might, perhaps, be aroused enough to answer one question, but would relapse immediately into a snoring sleep.

10. Deafness was almost unfailing, and to such a degree, in some cases, as to render it difficult to make the patient hear.

11. Retention of urine was an occasional source of trouble, most common in females.

12. Forgetfulness of what had occurred during the disease was common in the severe cases, and in the mild the memory was confused. Unconsciousness of the lapse of time may be mentioned in this connection; and, if a patient was unable to tell at any time how long he had been sick, or could not remember the day of the week, the prognosis might be considered as doubtful.

13. The rose-colored eruption attended a sufficient number of cases to be considered as diagnostic.

14. Subsultus and hiccough also were common—the former in cases of every grade, but the latter only in the worst cases.

Respecting the nature of this disease there can be no doubt. The foregoing enumeration of symptoms render its peculiar character conclusive.

It may be well to mention some facts connected with the prevalence of this endemic, which will be interesting. The starch-mill is situated at the southern extremity of Main-street, which extends north and south. Crossing Main-street at right angles, about eighty rods north of the mill, is another street (St. Clair). There is a ravine commencing at the mill, which extends to the N.E., crossing St. Clair-street about thirty rods east from the corner. In a north-west direction from it also, is a small stream crossing St. Clair-street, eighty rods from the corner. The man who died from congestion of the brain lived in a north-west direction from the mill. Two or three of his children had the disease, one of whom died, having all the appearances peculiar to it. During the month of November the wind was mostly north-east, carrying the effluvium in the direction of this family, and the smell was noticed more than a mile west from the mill. The prevailing wind after Winter set in, was S.W., which would carry the miasm in the ravine first mentioned; and it was in that part of the village crossed by this ravine that the majority of cases occurred. The first fatal case, and almost the first one that occurred after the commencement, was that of the young lady who, after seventeen days from the accession of typhoid symptoms, was attacked with hæmorrhage from the bowels, and died in a short time.

The smell of the potatoes was noticed little or none in parts of the village directly north from the mill, while N.E. and N.W. it was almost intolerable. The subjects were generally young persons from eight to thirty years of age. In some instances, whole families were affected. Facts may be related which would lead us to consider the disease as contagious. About the time that Miss B. (the young lady referred to) died, a daughter of Mr. K., who lived in the house adjoining, was attacked. About the same time, a sister of Miss B. was attacked, and died on the seventeenth day of the disease. A young lady (Miss F.) who assisted in the family of Mr. K. was also taken sick, and remained in the family. A son of Mr. K. was taken about ten days after the daughter, and Mrs. K. was attacked before the rest recovered. After the younger Miss B. died, and previous to the attack of the other, the family removed to a house across the street. The wife of the man who went into the house Mr. B. had left, was attacked about the same time. Her husband also had some of the symptoms, but they were thrown off. Miss A., who went into the family of Mr. K. after Miss F. was taken sick, began to complain in about a week, and went home to an adjoining town ten miles distant. She had the disease and died. Three or four of her father's family were afterwards attacked, and a brother died. No other cases occurred in that neighborhood. A sister-in-law and niece of Mr. K., who had assisted considerably in the care of the sick in his family, had the disease, but in a mild form. They resided on Main-street, out of the range of the miasm, and were not in Mr. K.'s house more than one-third of the time. Mr. K. had some of the premonitory symptoms, but the disease was not developed. A sister of Miss F., who was with her about four weeks, was attacked and went home. She had the disease, and a brother, who resided in the family, also took it from her and died. A young man who resided in the family was attacked, and before he recovered, his father, who resided about twenty-five miles south, came and took him home. After his arrival at his father's, some of the family was taken sick, and from thence the disease spread until the whole household (the parents and seven children) died; the one who was taken sick here alone escaping.

After the death of the second Miss B., her father, a sister, and three other members of the family were attacked, and had a severe form of the disease. Many others of the neighbors who had watched with these families, either had the disease or felt the symptoms.

Some cases occurred in parts of the village upon which the miasm was not wafted by the wind; but in every instance, we think, the subjects had assisted more or less in the care of the sick in the infected district. These cases were of the middle or mildest grade, and the disease was not communicated from them to others. Those who spent only part of the time with the sick were affected much less, provided the balance was passed in parts not visited with the disease. Those who washed the clothes used about the sick, or devoted all their time with them, were most exposed.

Another fact is worthy of mention. The persons who worked in the mill until the potatoes that did not rot were all converted into starch, were not subjects of the disease, unless they spent their time, when not at work, in the infected district. There were a number of families residing within a few rods of the mill, who were untouched. We may infer from this, that miasm, to produce any effect upon the health, should be diffused to a certain extent through the atmosphere. That this idea is correct will be shown by other facts.

The above remarks apply to the disease as it prevailed during the Winter of 1846 and 1847.

After the opening of Spring, no cases occurred until September, when the disease reappeared. In the main, it presented the same characteristics during the autumnal months, as it had during the previous Winter. The principal variation was a tendency to assume a periodical form after the crisis, there being a chill, succeeded by the hot and sweating stages, as in ordinary intermittents, and susceptible of being arrested by the prompt administration of anti-periodics.

In such cases there seemed to be, in addition to the local miasm, the usual general influence tending to produce intermittents; and the typhoid tendency predominating, and expending its force first, left the system a prey to a predisposition less potent. This fact goes to prove that more than one cause may be operating in the atmosphere at the same time, each in its turn producing its effect according to its power to do so.

Such results occurred in cases where there was no organic lesion in any part, but a debilitated condition of the system, produced by the effect of the continued form of fever. When the crisis arrived the system was in a state to convalesce; but the ordinary causes of intermittents being in operation, a paroxysm would come on as stated above. Recovery would follow rapidly after the periodic tendency was interrupted, as after ordinary intermittents.

A majority of the cases that occurred during the Autumn of 1847, were in individuals who had worked in and about the starch mill, and in families residing on Main-street near the mill. The smell was not so intense as to be noticed at any distance from its source, as the pile of potatoes had diminished to a great extent. From this, too, we see that miasm must be diluted and diffused to a certain degree through the atmosphere, to be operative. At this time, the emanation was just sufficient to produce the disease in those nearest the source.

The number of fatal cases was small, the proportion not being any higher than in our usual autumnal fevers, although in many instances the disease was exceedingly severe and malignant. When once established, it could not be interrupted; still, by proper management, its violence could be lessened, and the patient enabled to pass the crisis without fatal sinking. In cases where the diarrhœa was not very profuse, and could be controlled to a certain degree by medicine; the delirium not excessive, a favorable result generally followed.

The most unfavorable symptoms were profound coma, or raving delirium; involuntary evacuations; hæmorrhage from the bowels; cold, clammy sweats; with a pulse more than 135 in a minute. In many cases the pulsations were as many as 140 or 150 in the minute, and the patient recovered; but in one instance only do I recollect of an adult recovering, whose pulse was higher than 160.

Cases in which the pulse was not over 110, but very feeble, with a pale, flabby appearance to the tongue, great prostration of strength, subsultus, hiccough, &c., were considered in great danger; but many recovered in which the whole catalogue of unfavorable symptoms existed. Persons of intemperate habits, and those whose constitutions had suffered from other depressing causes, were the greatest sufferers.

Post mortem examinations were made in three cases. The first was in that of the young lady first mentioned. There were ulcerations through the whole extent of the intestines, from the duodenum to the rectum, the deepest being in the small intestines. The ulcerated patches were from the diameter of two lines, to that of a pin head. The intestines were found filled with black blood, and before death some quarts had passed off. She might be said to have died of hæmorrhage, as the other symptoms had not been particularly unfavorable. She, it will be remembered, died in December, 1846.

The second was in the case of a little girl, four years of age, who died in the Fall of 1848. She had had whooping-cough for two or three weeks at the time the fever set in, and her death was caused by congestion of the brain. The brain was filled with blood, and the lungs also were considerably congested. The mucous membrane of the small intestines was inflamed to a considerable extent. There were small elevated spots upon the internal surface of the bowels, which were of a bright red. This case was an interesting one, from the fact that we had an opportunity of examining the condition of the entire mucous membrane at an early stage of the disease, which we could seldom have done, as the fever was not a sufficient cause to produce death. There was much more disease than any external signs indicated, from which we might infer that in all cases there was inflammation to a greater or less extent.

The third case was that of a young man twenty-two years of age, who died in the autumn of 1847. He had resided during the summer in St. Joseph's County, and had suffered while there from a remittent fever. At the time he was attacked with continued fever, he had not recovered from his former sickness, but was able to be about the streets. He was not as sick to appearance as many others had been who recovered. He died on the twenty-second day from the time of attack. Half-an-hour before his death, he asked to be helped into a chair, was cheerful, spoke of his hopes of recovery, and took some food. He very soon complained of a severe pain in the abdomen, and a deathly faintness. Stimulants were administered, but he soon sunk and died in the chair. In this case there were extensive ulcerations of the small intestines, throughout the whole extent. About a foot of the ileum was gangrenous, with perforations in two or three places, the largest of which was an inch and a half in length. The intestines were diseased through the whole length to some degree, presenting a livid appearance, and considerable effusion had taken place in the abdominal cavity. He had strength enough to walk across the room within five days of his death.

No uniform course could be pursued in the treatment of this disease, it being necessary to vary according to the severity of the attack, and the grade of type assumed. In the mild cases, if called at an early stage, a laxative to remove morbid matter or fæcal accumulations was advisable.

In a majority of cases, the disease had advanced so far before medical aid was called, that this was inadmissible. Frequently I have known a free dose of calomel given, when the first symptoms made their appearance, with the effect to completely break up the disease. Sometimes blue pill, or alterative doses of calomel, at the outset were beneficial; but, generally, mercury in any form failed to produce any good results,—and when given so as to produce constitutional effects, positive injury was done, as it increased nervous irritability and general depression.

As alteratives and sedatives were indicated, a favorite prescription with me was as follows :—

| | |
|---|---|
| ℞ Mur. Ammoniæ, | ʒij. |
| Fol. Digitalis, | ʒj. |
| Pulv. Ipecac., | grs. xxv. |
| Sacch. Alb., | q. s. |

The digitalis and ipecac. to be infused in four or five ounces of boiling-water; after which add the muriate ammonia, and sugar enough to sweeten the compound. A teaspoonful to be given in half an ounce of some liquid once in four hours.

This preparation was varied according to circumstances. The ipecac. was sometimes diminished or left out, and where there was much vascular debility and the pulse not very frequent, the digitalis was omitted. The muriate of ammonia was generally tolerated, but the addition of some aromatic would prevent nauseating effects.

Alternated with the above-mentioned preparation, opium either alone or in conjunction with ipecac., was used when there was looseness of the bowels; and, in some cases, acetate of lead, kino, or catechu were found necessary. Sponging the body with water at a temperature most agreeable to the patient's feelings, afforded considerable relief. Cold applications to the head were beneficial, when there was local determination producing nose-bleed, &c. Diaphoretics and diuretics, according to the circumstances of the case, together with such other means suggested by general principles, made up the treatment for the first week after the accession of the disease. As the tongue became dryer and the diarrhœa more profuse, oil turpentine in doses of from six to ten drops once in six hours, was a valuable remedy in conjunction with opium, &c. Sinapisms and fomentations to the abdomen, when there was tenderness and tympanites, were beneficial; and also oil of turpentine applied as a rubefacient. Blistering the epigastrium and abdomen was attended with good results in many cases. In cases attended with nervous irritability, an infusion of valerian was useful, given in free doses. It would frequently produce sleep where there was morbid watchfulness, especially in females of an extremely nervous temperament, who would not tolerate opium. An ethereal tincture of castor would also produce the same effect.

At the commencement of the second week, as the symptoms became aggravated and the patient weaker, muriate ammonia alone, in conjunction with turpentine and opium if indicated, were principally employed. It was sometimes necessary to employ some mild tonic in the worst cases, and serpentaria was found to serve such a purpose very well.

Cold drinks were generally preferred, and a small quantity of cold water or elm-water, often repeated, was the most acceptable. Along with medicines, a proper use of some mild diet was deemed important; and it was found best to administer a small quantity of nourishment as regularly as the medicines were given. In the mild cases, some simple gruel was found sufficient, made of flour or rice boiled very soft. In the severe cases, attended with debility, beef-tea or animal broths were found necessary. During the convalescence, an egg beaten up with brandy properly diluted, was a useful article of diet.

Inasmuch as there was such a dissimilarity in the different cases as to severity, modifying symptoms, and local complications, it was impossible to follow any one mode of treatment. Each case had to be managed according to its own merits; and my own views upon this subject cannot be more concisely expressed than by quoting the remarks of Dr. Watson (Op. Cit., edition of 1851, p. 963.) "Summarily expressed," he says, "it consists in the exercise of incessant vigilance, and the adoption of the proper remedy at the proper moment. It lies between a timid or sceptical abandonment of all known resources, and a meddlesome rashness in applying them. The flame of life may be suffered to expire without timely succor, by the practitioner who folds his arms and looks on; as it may be rudely extinguished by a restless or routine interference, which has no definite or intelligible purpose."

The convalescence, in different cases, depended upon the extent of injury, both functional and organic, which the system had sustained. If there were no organic lesion, and the patient of a good constitution, recovery was rapid. When the disease had expended its force, all the functions seemed to resume their office, and the individual became renewed in physical energy. In some cases, those who had suffered were more robust than before their sickness.

The disease in question continued to prevail till the Winter of 1847–48, when its violence was stayed. Since then, however, there have been more or less cases, until the present time, varying in severity from year to year. Intermittents and remittents have been less frequent, and when they do occur there is a tendency to assume a typhoid type. Less active treatment is necessary in the management of all diseases.

In reviewing the phenomena manifested in this endemic, we find some facts not hitherto mentioned that are interesting. One is, that miasm will follow the course of streams, or the lowest places in the land. The smell from the pile of potatoes was very distinct along the course of the stream and ravine mentioned; whereas, in other directions and localities it was hardly noticed.

Another fact was, that the disease was most prevalent in those parts upon which the wind blew most constant at the time.

Another still may be mentioned. It was found that the pulse of persons in health, who had resided in the infected district, was increased in frequency, in more than one instance being one hundred in a minute. From this it seems that there was a general predisposition to the disease, though not developed in every case.

A variety of causes operated to change the predisposition to the disease itself; and those who were most with the sick, and who violated to the greatest degree the laws of health, were its subjects.

Almont, Lapeer Co., Mich., *December*, 1852.

